# Elevated Levels of Dickkopf-1 Are Associated with β-Catenin Accumulation and Poor Prognosis in Patients with Chondrosarcoma

**DOI:** 10.1371/journal.pone.0105414

**Published:** 2014-08-21

**Authors:** Changbao Chen, Hua Zhou, Xiaolin Zhang, Xinlong Ma, Zhongjun Liu, Xiaoguang Liu

**Affiliations:** 1 Department of Spinal Surgery, Tianjin Hospital, Tianjin, China; 2 Department of Orthopaedic Surgery, Peking University Third Hospital, Beijing, China; University of Alabama at Birmingham, United States of America

## Abstract

**Background:**

Dickkopf-1 (DKK1) is an antagonist of Wnt/β-catenin signaling implicated in tumorigenesis. However, the biological role of DKK1 and β-catenin involved in chondrosarcoma has not been sufficiently investigated. This study was designed to investigate the expression profiles of DKK1 and β-catenin, and to clarify their clinical values in chondrosarcoma.

**Methods:**

The mRNA and protein levels of DKK1 and β-catenin in fresh chondrosarcoma and the corresponding non-tumor tissues were analyzed by Real-time PCR and Western blot, respectively. The protein expression patterns of DKK1 and β-catenin were investigated by immunohistochemistry. The associations among DKK1 level, β-catenin accumulation, clinicopathological factors and the overall survival were separately evaluated.

**Results:**

Both DKK1 and β-catenin levels were remarkably elevated in chondrosarcoma compared with the corresponding non-tumor tissues. High DKK1 level and positive β-catenin accumulation in chondrosarcoma specimens were 58.7% and 53.9%, respectively. Elevated DKK1 level significantly correlated with positive β-catenin accumulation, and they were remarkably associated with histological grade and Musculoskeletal Tumor Society stage. Furthermore, DKK1 level and β-catenin accumulation had significant impacts on the prognosis of chondrosarcoma patients. Multivariate analysis revealed that DKK1 level was an independent prognostic factor for overall survival.

**Conclusions:**

Elevated DKK1 levels associated with β-catenin accumulation play a crucial role in chondrosarcoma. DKK1 can serve as a novel predictor of poor prognosis in patients with chondrosarcoma.

## Introduction

Chondrosarcoma is a malignant cartilage-forming tumor, accounting for approximately 20% of bone malignancies and presenting with a wide spectrum of clinical behaviors [1]. Since conventional chondrosarcoma is highly resistant to chemotherapy and radiotherapy [2], surgical resection remains the important treatment option. Despite all-out efforts to prevent the recurrence and progression of this malignancy, it was accompanied with a significant rate of morbidity and a 10-year survival rate of 29–83% [2], [3]. Therefore, it is an urgent need to prevent recurrence or treat inoperable micro-lesions by exploring novel biological targets for future tailored therapy based upon the molecular events underpinning the biology of chondrosarcoma [4].

The WNT signal transduction cascade is implicated in several important physiological and pathophysiological conditions such as tumorigenesis [5]. In the canonical Wnt/β-catenin signaling model, Wnt protein bind to members of the Frizzled family and the transmembrane protein LRP5/6 to prevent phosphorylation and degradation of β-catenin by the GSK3β/APC/Axin destruction complex. Subsequently, accumulated nuclear β-catenin bind to the members of LEF/TCF transcription factor family activating gene expression programs [6]. Wnt also activates the so-called non-canonical signaling that regulates the planar cell polarity, and provokes calcium mobilization probably [7]. The canonical Wnt/β-catenin signaling is intricately regulated at many levels, including by the Dickkopf protein family [8], [9]. The Dickkopf (DKK) genes were Wnt antagonists originally identified as inducers of head formation in Xenopus and comprised an evolutionary conserved gene family of four members (DKK1-4) and a unique DKK3-related gene, soggy [9], [10]. Recent biochemical and genetic studies have argued that DKK1, as an Wnt antagonists, disrupts Wnt-induced Fz-LRP6 complex formation and inhibits canonical Wnt signaling [5], [6], [11]. In addition, DKK1 was identified as a downstream target of the β-catenin/TCF pathway and participates in a negative feedback loop in the Wnt signaling pathway [12]. These finding suggest that DKK1 associated with β-catenin accumulation plays an important role in cancerogenesis [13].

Abnormalities in Wnt/β-catenin signaling are implicated in a variety of human cancers [5], [6]. DKK1 is typically silenced in colon cancer and acute myeloid leukaemia (AML) by DNA hypermethylation that correlated with advanced stages of colorectal tumorigenesis and may be a useful prognostic marker in AML [14], [15]. The expression levels of DKK-1 are also reportedly downregulated in melanoma cells and colorectal cancer [16], [17]. In contrast, DKK1 is reported to be overexpressed in many malignant tissues including breast cancer, lung and esophageal carcinomas, multiple myeloma, hepatocellular carcinoma (HCC), and osteosarcoma [18]–[22]. Moreover, DKK-1 can serve as a novel diagnostic and prognostic biomarker [19], [21], [22] and may have roles in bone metastases of breast and prostatic carcinomas [23], [24]. Therefore, the function and role of DKK-1 in cancer appears to depend on the cancer cell type and the tumor microenvironment. Accumulated observations have implied that cytoplasmic and/or nuclear β-catenin accumulation is detected in many solid tumors and can be predictive for poor prognosis including sarcoma [21], [25]–[27]. We have previously documented that HIF-1α and HIF-2α play central roles in the pathogenesis of chondrosarcoma, and can be predictive for the prognosis of chondroarcoma patients [28], [29]. More importantly, HIF-1α can trigger nuclear translocation of β-catenin in hypoxia [30]. These reports have implied that deregulation of Wnt/β-catenin signaling is a frequent event in tumor biology and may be implicated in the development and progression of chondrosarcoma.

The potential association of deregulation of Wnt/β-catenin signaling with chondrosarcoma development provides the rationale for deeply exploring the underlying role of DKK1 and β-catenin in chondrosarcoma in vivo, and determines whether DKK1 and beta-catenin can be predictive for overall survival of patients with chondrosarcoma. Our findings suggest that elevated levels of DKK1 and β-catenin play a role in the pathogenesis of chondrosarcoma, and DKK1 can be recognized as an independent factor to predict the prognosis of chondrosarcoma patients. Thus it could be a potential biological target for the management of chondrosarcoma.

## Materials and Methods

### Ethics Statement

Signed informed consent from all patients was obtained for sample collection and analysis. All samples were anonymously coded in accordance with local ethical guidelines. This study was approved by the institutional review board of the Peking University Third Hospital and Tianjin Hospital and was in full compliance with national legislation and the ethical standards of the Chinese Medical Association (IRB00001052-08044).

### Clinical Specimens

Sixty-three cases of conventional chondrosarcoma, seventeen cases of benign cartilage tumors including osteochondromas and enchondromas based on accepted clinicopathological and radiological criteria were enrolled in this study [31]. We excluded rare subtypes of chondrosarcoma such as dedifferentiated, mesenchymal, juxtacortical, and clear-cell chondrosarcoma. Fresh chondrosarcoma tissues and the corresponding non-tumor tissues were carefully selected from the department of orthopaedics in Peking University Third Hospital. Routinely fixed paraffin-embedded tumor specimens were obtained from the department of pathology, Peking University Third Hospital and Tianjin Hospital. Histological grading and surgical staging system of the Musculoskeletal Tumor Society (MSTS) were carefully evaluated as reported previously [28], [29]. The time of follow-up was calculated from the date of surgical operation. The median time of follow-up was for 30 months (4 to 98 months).

### Quantitative Real-time PCR (qPCR)

RNA extraction, cDNA synthesis, and quantitative polymerase chain reactions (PCR) were performed as described previously [29]. Briefly, total RNA was extracted from chondrosarcoma samples and the corresponding non-tumor tissues with TriZol reagent (Invitrogen). First-strand cDNA was generated using the total RNA in a reverse transcriptase reaction using a poly (dT) oligonucleotide as a primer and SuperScript II reverse transcriptase (Invitrogen). cDNAs were then subjected to quantitative real-time PCR analysis. Quantitative real-time PCRs were carried out using SYBR Green PCR Master Mix (Applied Biosystems). Two oligomers of primers were synthesized based on the reported sequences of Dikkopf-1 (5′-GATCATAGCACCTTGGATGGG-3′ and 5′-GGCACAGTCTGATGACCGG-3′) [17], β-catenin (5′- AGCCGAGATGGCCCAGAAT-3′ and 5′-AAGGGCAAGGTTTCGAATCAA-3′) [32], and GAPDH (5′-ATCATCCCTGCCTCTACTGG-3′ and 5′-CCCTCCGACGCCTGCTTCAC-3′). PCRs were optimized for the number of cycles to ensure product intensity to be within the linear phase of amplification. The cycles were as follows: 95°C for 10 min followed by 40 cycles of 95°C for 15 s, 55°C for 35 s and 72°C for 30 s. Data were analyzed according to the relative standard curve method with normalizing the values of GAPDH expression in each sample.

### Western Blot

Protein extraction and Western blot were performed as previously described [28]. Primary antibodies were as follows: anti-actin mouse monoclonal antibody (1∶1000; Sigma), anti-Dkk-1 rabbit monoclonal antibody (1∶1000; Abcam), and anti-β-catenin mouse monoclonal antibody (1∶1000; Transduction Laboratories). Bound antibody was detected using an appropriate IRDye 700 or 800-conjugated secondary antibody against mouse or rabbit IgG (LI-COR Bioscience, USA), and visualized using infrared fluorescence. Images were obtained using the Odyssey Infrared Imaging System from LI-COR Biosciences.

### Immunohistochemistry and Evaluation of staining

Detailed experimental procedures for immunohistochemistry have been described previously [29]. Briefly, all specimen slides (5µm) were deparaffinized and rehydrated, and then endogenous peroxidase activity was blocked with hydrogen peroxide. After antigen retrieval, DKK1 (1∶250; Abcam) and β-catenin (1∶500, clone 14; Transduction Laboratories) immunoreactivities were detected using the EnVision+, Peroxidase system (DAKO Diagnostics). The slides were then developed with DAB substrate (DAKO Diagnostics) and counterstained with hematoxylin. Negative controls were included by omitting primary antibody replaced by PBS.

All the stained slides were simultaneously scored blinded by two, independent observers without prior knowledge of clinicopathologic data, and a consensus score was reached for each slide. For the assessment of the intensity of DKK1 staining, DKK1 staining was evaluated using the following criteria as described previously [19]: Strong positive (scored as 2+), dark brown staining in >50% of tumor cells completely obscuring cytoplasm; Weak positive (1+), any lesser degree of brown staining appreciable in tumor cell cytoplasm; and Absent (scored as 0), no appreciable staining in tumor cells. For all analysis, strongly positive staining (scored as 2+) was identified as high level; otherwise, the tumor was considered as low level. In particular, immunopositive scoring results for nuclear accumulation of β-catenin were available as reported previously [25]. The extent of the nuclear staining was measured carefully as follows: No reactivity, 0 (negative); Nuclear reactivity in up to 10% of cells, 1+ (weak); Nuclear reactivity 10–50% of cells, 2+ (moderate); Nuclear reactivity in >50% of cells, 3+ (strong). For the evaluation of β-catenin protein immunoreaction, we considered more than 10% positive nuclei of cells involved per tissue sample as positive results (nuclear accumulation); otherwise, the tumor was considered as negative results.

### Statistical Analysis

Data are presented as the Mean ± SD. Differences between groups were analyzed using the Student’s t-test for continuous variables, or the Pearson’s Chi-square test/Fisher’s Exact Test for categorical variables. Curves for overall survival were drawn according to the Kaplan-Meier method, and differences between the curves were analyzed by applying the log-rank test. Univariate and multivariate analyses of clinicopathological factors and overall survival were done using Cox proportional hazards regression model. Statistical analyses were performed using SPSS (SPSS, Chicago, IL, USA.) and *P*<0.05 was considered statistically significant. All reported *P* values were two-sided.

## Results

### Elevated levels of DKK1 and β-catenin accumulation in chondrosarcoma tissues

**Figure 1:**
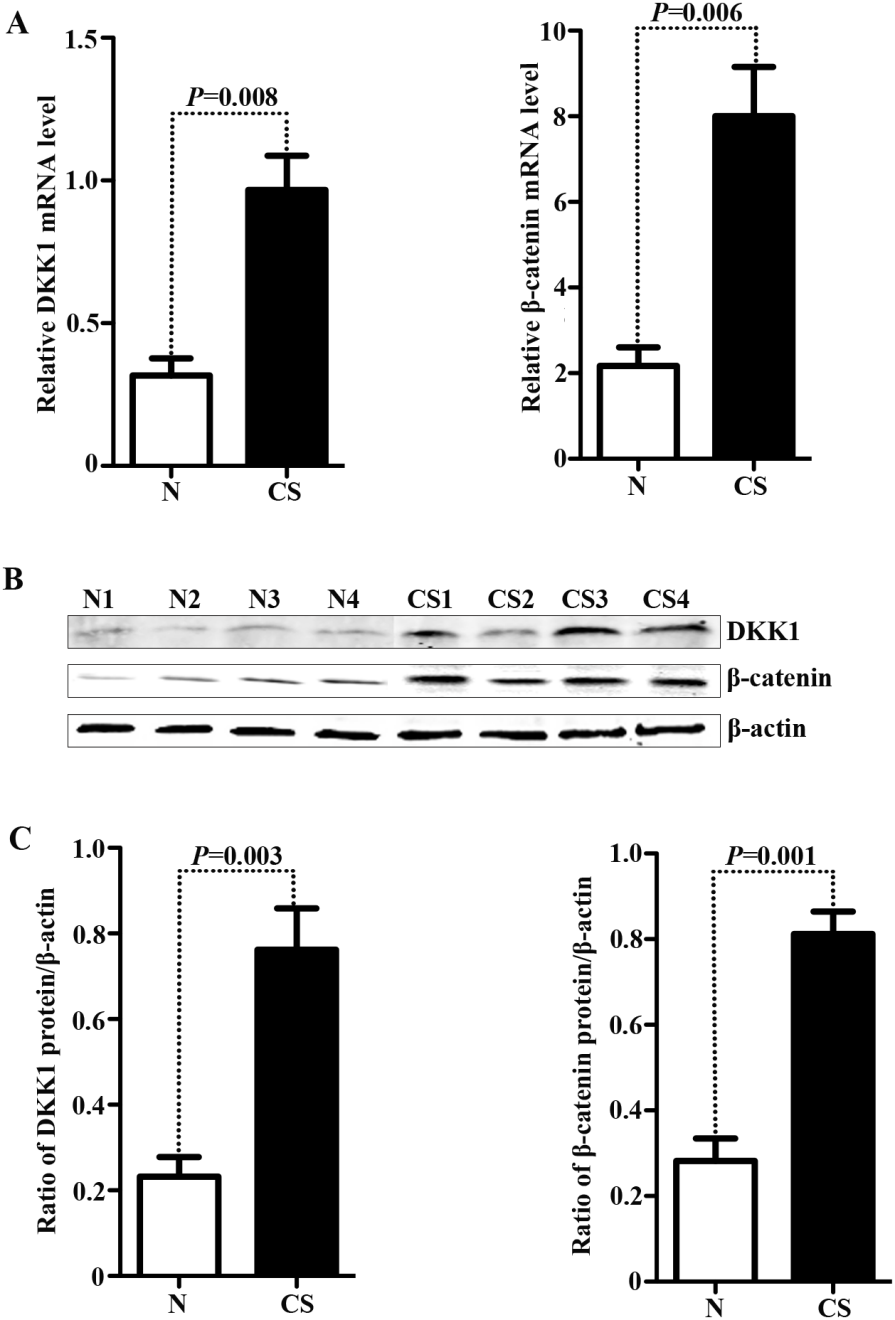
Expression levels of DKK1 and β-catenin in human chondrosarcoma tissues. (**A**) The mRNA levels of DKK1 (*left panel*, *P* = 0.008) and β-catenin (*right panel*, *P* = 0.006) were analyzed by Real time PCR in eight chondrosarocma tissues and the paired corresponding non-tumor tissues. N, the corresponding non-tumor tissues; CS, chondrosarcoma tissues. (**B**) The protein levels of DKK1 and β-catenin were detected by Western blot. To confirm equal protein levels, the same blot was stripped and developed with anti β-actin antibody. Four representative pairs of tissues were presented. Representative results of three experiments performed were shown. (**C**) The diagram provided represented the statistical analysis of DKK1 (*left panel*, *P* = 0.003) and β-catenin (*right panel*, *P* = 0.001) of eight paired cases.

**Figure 2:**
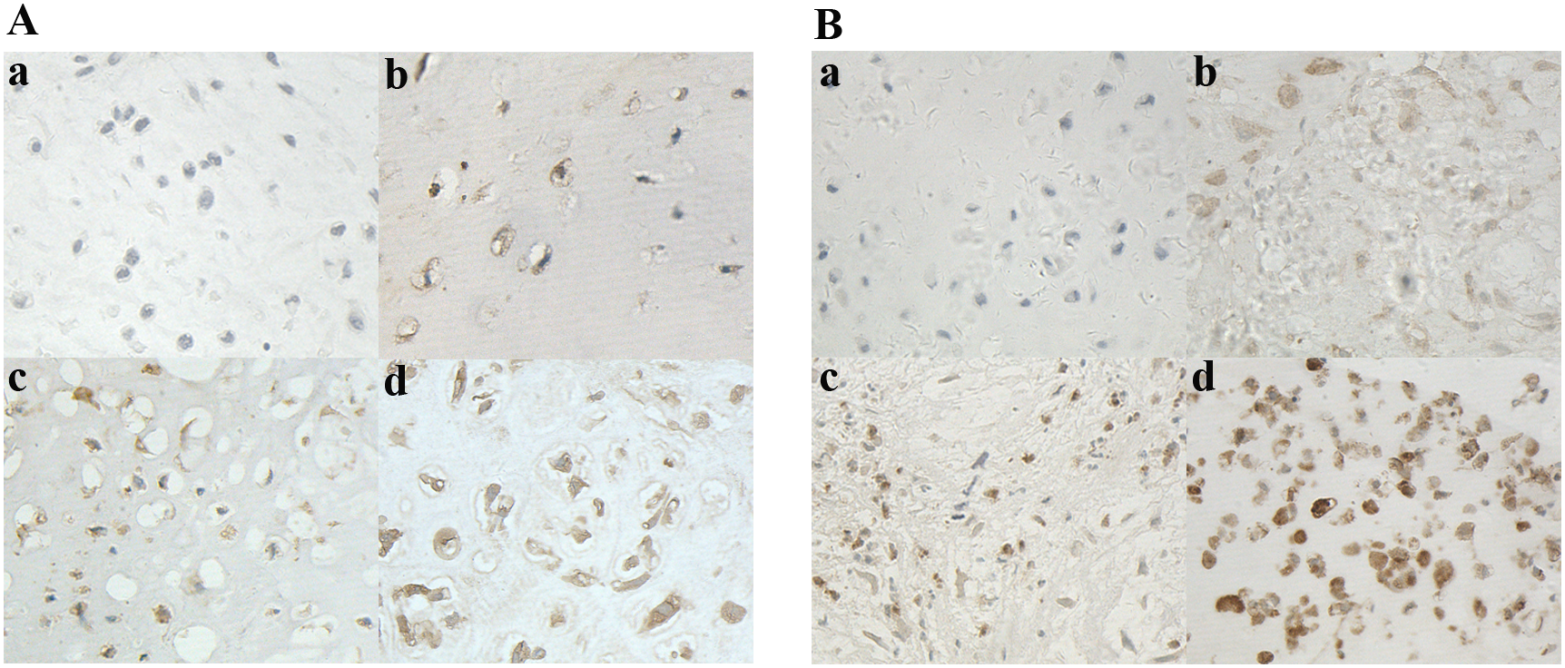
Representative images of DKK1 and β-catenin immunohistochemical staining in human cartilage tumors. (**A**) The expression profiles of DKK1 staining are shown in human cartilage tumors. Negative cytoplasmic DKK1 staining was observed in enchondroma (**a**). A well-differentiated (Grade I) chondrosarcoma with negative cytoplasmic DKK1 expression (**b**). A moderately differentiated (Grade II) chondrobsarcoma with weak cytoplasmic DKK1 expression (**c**). A poorly differentiated (Grade III) chondrosarcoma with prominent and strong cytoplasmic DKK1 expression (**d**). (**B**) The expression patterns of β-catenin are presented in human cartilage tumors. Enchondroma with negative β-catenin immunostaining (**a**). Weak (**b**), moderate (**c**), and strong (**d**) nuclear immunostaining of β-catenin accumulation were detected in chondrosarcoma, respectively. Original magnification×400.

To identify whether the expression levels of DKK1 and β-catenin are involved in the pathogenesis of chondrosarcoma, we measured the mRNA and protein levels of DKK1 and β-catenin in chondrosarcomas and the corresponding non-tumor tissues. Our results shown that the mRNA levels of DKK1 and β-catenin in chondrosarcomas were significantly increased compared with the corresponding non-tumor tissues (*P* = 0.006, Fig. 1); Similarly, a remarkable elevation in DKK1 and β-catenin protein was also observed in chondrosarcoma as compared to the corresponding non-tumor tissues (Figure 1; *P* = 0.001, Figure 1). To explore the expression profiles of DKK1 and β-catenin in human cartilage tumors, we performed immunochemistry analysis for 63 patients with chondrosarcoma and 17 patients with benign cartilage tumors. As shown in Figure 2A, the positive signal of DKK1 was preferentially identified at the cytoplasm, and high DKK1 expression was 58.9% (37/63) in chondrosarcomas samples as compared with 23.5% (4/17) in benign cartilage tumors (*P* = 0.014, Table 1). The immunoreactive signal of β-catenin was predominately distributed at the nucleus (Figure 2A), which is consistent with the previous report [26], [27], and positive β-catenin accumulation was considered as 53.9% (34/63) in chondroarcoma specimens as compared to 17.6% (3/17) in benign cartilage tumors (*P* = 0.012, Table 1). These findings suggest that elevated levels of DKK1 and β-catenin accumulation may contribute to the pathogenesis of chondrosarcoma in vivo.

**Table 1:** Expression level of DKK1 and β-catenin accumulation in human cartilage tumors.

Groups	Total No. of cases	DKK1 Level		*P-*value	β-catenin Accumulation		*P-*value
		Low	High		Negative	Positive	
Benign cartilage tumors	17	13	4	0.014*	14	3	0.012*
Chondrosarcomas	63	26	37		29	34	

*P* values recorded are the results from Fisher’s Exact Tests. *P<0.05.

### Association of DKK1 level, β-catenin accumulation, and clinicopathogical factors

The associations among DKK1 level, β-catenin accumulation and clinicopathological factors are presented in Table 2. DKK1 level significantly increased with the advancement of histological grade (*P* = 0.035) and MSTS stage (*P* = 0.020). Similarly, β-catenin accumulation was markedly correlated with advanced clinicopatholgoical parameters such as higher histological grade (*P* = 0.037) and higher Musculoskeletal Tumor Society stage (*P* = 0.011). Furthermore, we found that both DKK1 level and β-catenin accumulation had no significant correlation with other clinicopathological factors such as gender, age and anatomical location.

**Table 2:** Association among DKK1 level, β-catenin accumulation and clinicopathological factors in 63 patients with chondrosarcomas.

Clinicopathological Factors	Total No. of cases	DKK1 Level		*P-*value	β-catenin accumulation		*P-*value
		Low	High		Negative	Positive	
Gender				1.000			0.610
Male	36	15	21		18	18	
Female	27	11	16		11	16	
Age(years)				0.362			0.769
≥40	49	22	27		22	27	
<40	14	4	10		7	7	
Anatomical Location				0.279			1.000
Limb bone	21	11	10		10	11	
Axial bone	42	15	27		19	23	
Histological Grade				0.035*			0.037*
Well-differentiated (I)	32	18	14		19	13	
Moderate (II)	19	6	13		8	11	
Poorly (III)	12	2	10		2	10	
MSTS stage				0.020*			0.011*
I A + I B	34	19	15		21	13	
II A+ II B	29	7	22		8	21	
β-catenin accumulation				0.000*			
Negative	29	20	9				
Positive	34	6	28				

*P* values recorded are the results from Chi-square Tests or Fisher’s Exact Tests. *P<0.05.

### Relationship between DKK1 level and β-catenin accumulation in chondrosarcoma

Previous studies have documented that DKK1 was significantly associated with β-catenin expression in other tumors [21], [33]. Thus, it is pertinent at this point to determine whether DKK1 level correlates with β-catenin accumulation in chondrosarcoma. We found that 37 cases showed high DKK1 level, in which the β-catenin–negative and –positive tumor frequencies were 24.3% (9/37) and 75.7% (28/37), respectively. By contrast, in the remaining 26 cases displaying low DKK1 level, 76.9% (20/26) were considered as β-catenin–negative, and 23.1% (6/26) were classified as β-catenin–positive. Our results shown that there was a strong positive correlation between elevated DKK1 level and positive β-catenin accumulation (*P* = 0.000, Table 2).

### The impacts of DKK1 level and β-catenin accumulation on prognosis of patients with chondrosarcoma

**Figure 3:**
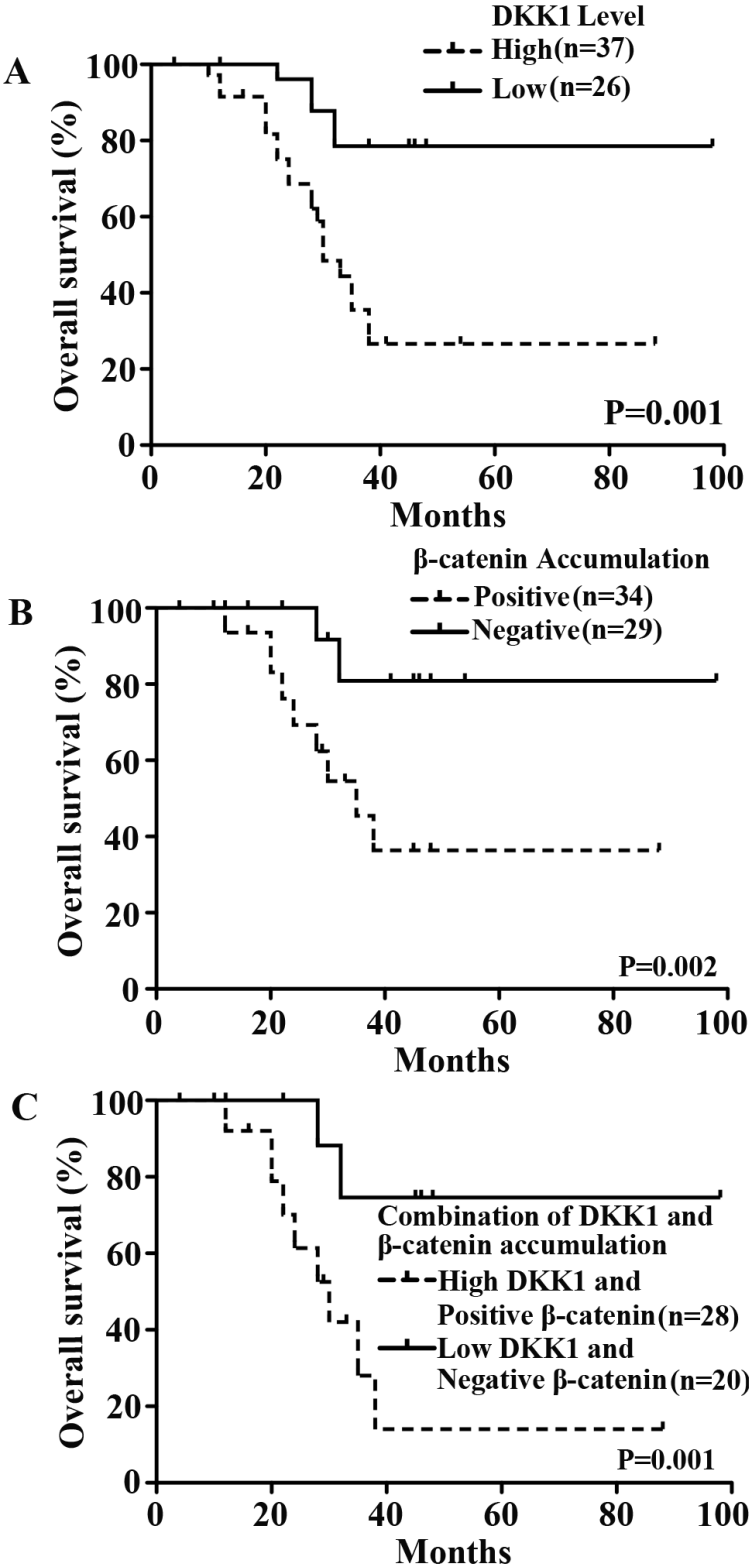
Prognostic value of DKK1 and β-catenin in 63 patients with chondrosarcomas by Kaplan-Meier survival curves and the log-rank test. (**A**) Probability of overall survival with regard to DKK1 level. High level of DKK1 protein was significantly associated with poor prognosis (*P* = 0.001). (**B**) Probability of overall survival with regard to β-catenin accumulation. Positive β-catenin accumulation remarkably correlated with poor prognosis (*P* = 0.002). (**C**) Probability of overall survival with regard to the combination of DKK1 level and β-catenin accumulation. Two groups were classified according to the expression level of DKK1 and β-catenin. The overall survival rate of patients with DKK1-high and β-catenin-positive tumors was significantly worse than that of patients with DKK1-low and β-catenin-negative tumors (*P* = 0.001).

Previous studies have documented that DKK1 and β-catenin can be predictive for the prognosis of patients with several solid tumors [19], [21], [22], [25]–[27]. Thus, to determine whether DKK1 level and β-catenin accumulation could be predictive for the prognosis of 63 cases of chondrosarcoma patients, the Kaplan-Meier analysis was performed, and showed that DKK1 level had a significant effect on the overall survival (*P* = 0.015, Figure 3A). Similarly, the survival rate of patients with β-catenin-positive tumors was significantly lower than that of patients with β-catenin-negative tumors (*P* = 0.018, Figure 3B). Furthermore, the combination of DKK1 level and β-catenin accumulation had a significant impact on the overall survival (*P* = 0.010, Figure 3C).

To identify the potential independent and significant factors for the overall survival, we performed Cox regression analysis (Table 3). Univariate Cox regression analysis had identified that histological grade, MSTS stage, DKK1 level and β-catenin accumulation were significantly associated with the overall survival (*P* = 0.000, *P* = 0.003, *P* = 0.004, *P* = 0.005, respectively). In the multivariate Cox regression analysis, histological grade (HR, 2.913, *P* = 0.039), MSTS stage (HR, 12.809, *P* = 0.001) and DKK1 level (HR, 15.479, *P* = 0.003) had a significantly independent impact on patient prognosis, whereas β-catenin accumulation failed to demonstrate independency (*P* = 0.248).

**Table 3:** Results of univariate and multivariate Cox Regression analyses of the potential prognostic factors for overall survival in 63 patients with chondrosarcoma.

Clinicopathological factors	Unfavorable vs. Favorable	Univariate analysis		Multivariate analysis	
		Hazard Ratio	*P* value	Hazard Ratio	*P* value
Gender	Male vs. Female	1.281	0.582	1.325	0.643
Age	≥40 vs. <40	1.700	0.396	1.197	0.791
Anatomical location	Axial vs. Limb bone	1.298	0.588	2.472	0.170
Histological grade	Grade 3 vs. Grade 1 and 2	5.186	0.000*	2.913	0.039*
MSTS stage	II A+II B vs. I A+I B	3.818	0.003*	12.809	0.000*
DKK1 level	High vs. Low	4.936	0.004*	15.479	0.003*
β-catenin accumulation	Positive vs. Negative	4.767	0.005*	2.202	0.248

*P* values recorded are the results from Cox Regression Analyses. *P<0.05.

## Discussion

Our observations have implicated DKK1 and β-catenin in the pathogenesis and prognosis of chondrosarcoma. Elevated levels of DKK1 significantly correlated with nuclear β-catenin accumulation could be involved in the development of chondrosarcoma, and DKK1 can be identified as an independent prognostic factor for the overall survival in chondrosarcoma. These findings suggest that disruption of DKK1 suppressing nuclear β-catenin accumulation could be a novel attractive target for chondrosarcoma treatment.

Previous reports have demonstrated that DKK1 can suppress the Wnt-induced signaling that is frequently activated in tumor biology [34]. Consequently, the reduction and loss of DKK1 expression as a tumor-suppressive activity have been reported in human cancers such as malignant melanoma and colon cancer [16], [17]. However, DKK-1 is remarkably overexpressed in myriad tumor types including hepatocellular carcinoma and osteosarcoma [18]–[22]. Therefore, the diverse biological functions of DKK-1 in cancer biology appear to depend on the cancer cell types and the tumor niche. To our knowledge, we present the first evidence that the mRNA and protein levels of DKK1 were significantly elevated in fresh chondrosarcoma tissues compared with the corresponding non-tumor tissues. Moreover, malignant chondrosarcoma cells exhibited higher DKK1 levels, mainly in the cytoplasm, whereas benign cartilage tumors showed lower DKK1 levels (Figure 2A). Accumulated evidence has illuminated that DKK1 performs as an oncogenic factor rather than a tumor-suppressor in many tumors. Accordingly, elevated levels of DKK1 in chondrosarcoma would be consistent with a role for DKK1 in promoting development and aggressiveness of chondrosarcoma in vivo.

Aberrant activation of the Wnt/β-catenin signaling pathway has been implicated in tumorigenesis including sarcoma [26], [27]. Accumulated observations have implied that β-catenin accumulation is detected in many solid tumors and can be predictive for poor prognosis [25]–[27]. However, the underlying role of β-catenin in chondrosarcoma has not been sufficiently characterized. In the present study, we found that the mRNA and protein levels of β-catenin were remarkably increased in chondrosarcoma tissues compared with the corresponding non-tumor tissues (Figure 1). We also found that β-catenin was frequently expressed within the nucleus in chondrosarcoma cells (Fig. 3), which is line with other previous reports [26], [27]. Given the ability of β-catenin as oncogenic function, nuclear β-catenin accumulation could play a role in chondrosarcoma development. Previous studies have documented that during the peripheral chondrosarcoma progression of grade I towards grade III, IHH signaling gradually diminishes and Wnt signaling is lost [35]. Moreover, decreased Wnt signaling and increased TGF-β signaling in high-grade peripheral chondrosarcomas were reported by the same group [36]. Nevertheless, our findings do not support their suggestions, as 29 (53.9%) of chondrosarcoma cases were strongly nuclear β-catenin positive, while 14 (82.3%) of the benign cartilage tumors were nuclear β-catenin negative. Furthermore, β-catenin accumulation significantly increased with the advancement of histological grade (*P* = 0.035, Table 2). Accordingly, in agreement with the previous findings that cytoplasmic and nuclear β-catenin expression has been associated with a poorer prognosis in human cancers [25]–[27], we also found that nuclear β-catenin accumulation was significantly associated with poor prognosis in 63 patients with chondrosarcoma, although β-catenin accumulation failed to be identified as an independent prognostic factor for the overall survival by multivariate analysis. Limited numbers of cases and differences in the methods and chondrosarcoma tissues enrolled may explain the difference in nuclear β-catenin accumulation observed in these studies. Our study including 63 chondrosarcomas is an important addition to the existing literature, and our findings have confirmed that nuclear β-catenin accumulation is a common event in chondrosarcoma that could be implicated in the pathogenesis and prognosis of chondrosarcoma.

No reports have been documented regarding as the relationship between DKK1 levels and β-catenin accumulation in chondrosarcoma. In this study, elevated level of DKK1 positively correlated with β-catenin accumulation in chondrosarcoma tissues (*P* = 0.000, Table 2). As DKK1 has been identified as a downstream Wnt target gene [12], activated β-catenin signaling will provoke the upregulation of DKK1, thus this elevation of DKK1 may actually indicate the activation of the Wnt/β-catenin signaling pathway. These findings could also interpret the phenomenon of the significantly positive correlation between DKK1 level and β-catenin accumulation in chondrosarcoma tissues. Actually, we have conducted a series of experiments for activated Wnt signaling in chondrosarcoma, our results have revealed that recombinant DKK1 or overexpression of DKK1 can remarkably suppress the expression of active dephosphorylated β-catenin and TCF-reporter transcriptional activity in SW1353 chondrosarcoma cells (Figure S1). Thus, these findings can further interpret our results that DKK1 was elevated in chondrosarcoma tissues, and positively correlated with β-catenin in vivo. Moreover, the survival rate of the DKK1-high and β-catenin accumulation-positive group was remarkably worse than that of the DKK1-low and β-catenin accumulation-negative group (Figure 3C). These observations suggest that DKK1 and β-catenin proteins may play roles in the pathogenesis of chondrosarcoma, and are important factors significantly influencing the overall survival rate. Nevertheless, additional studies are needed to clarify the association and define their underlying role in the pathogenesis and progression of chondrosarcoma.

To date, only a few studies have reported the clinical or prognostic significance of DKK1 and β-catenin. Several authors have documented that DKK1 was preferential expressed in hormone-resistant breast tumors that was associated with poor prognosis [18] and had identified DKK1 as a serologic and prognostic biomarker for lung and esophageal carcinomas [19]. Nevertheless, they failed to identify it as an independent prognostic factor for these malignancies. A recent study has shown that high expression of nuclear β-catenin was strongly associated with poor prognosis and was an independent prognosticator for overall survival in non-small cell lung cancer [37]. However, the clinical significance of DKK1 and β-catenin in chondrosarcoma has not been well characterized. We found that both DKK1 and β-catenin were associated with histological grade and MSTS stage (Table 2). Subsequently, we further determined whether DKK1 and β-catenin had significant impacts on the overall survival. Our findings shown that DKK1 and β-catenin were significantly associated with the clinical prognosis (Figure 3). These findings are consistent with the previous reports that DKK1 and β-catenin can be predictive for the prognosis of patients with several malignancies [19], [21], [22], [25]–[27]. In particular, multivariate analysis had revealed that among the clinicopathological factors, DKK1 was identified as an independent prognostic factor for the overall survival in 63 patients with chondrosarcoma. More importantly, prior studies have demonstrated that exogenous expression of DKK1 increased the migration/invasion activity of mammalian cells, suggesting a significant role for DKK1 in progression of human cancer. Considered together, the above results and our findings suggest that DKK1 level seems to play an important role in the development and/or progression of certain types of human tumors including chondrosarcoma, although the link between DKK1 and Wnt signaling pathway in the context of chondrosarcoma development and progression remains under investigation at present. Therefore, high DKK1 level can be used as an indication for necessity of further adjunctive treatment. Moreover, these results might help the clinician to determine the prognosis of chondrosarcoma according to the levels of DKK1, and suggest the possibility of a novel biomarker of DKK1 in adjuvant therapy of chondrosarcoma with high DKK1 expression. To our knowledge, for the first time, we have explored the potential prognostic significance of DKK1 in chondrosarcoma. Because the sample size involved in this study may be small, further investigation of a larger patient population should be necessary to confirm its clinical value and prognostic evaluation in chondrosarcoma.

In conclusion, we have identified that elevated DKK1 levels associated with nuclear β-catenin accumulation play a role in the pathogenesis of chondrosarcoma, and can indicate poor prognosis of patients with chondrosarcoma. DKK1 has been proved to be an independently valuable factor in predicting the prognosis of patients with chondrosarcoma. The potential mechanism between elevated DKK1 expression and Wnt/β-catenin pathway activation and the mechanism by which the Wnt/β-catenin pathway is activated in chondrodarcoma development is still unknown. Through further understanding of the role of Wnt/β-catenin pathway activation, DKK1 and β-catenin would be a potential therapeutic target for chondrosarcoma management.

## Supporting Information

Figure S1 **DKK1 inhibition of canonical Wnt/β-catenin signaling in human chondrosarcoma SW1353 cells.** A. Soluble DKK1 inhibited active β-catenin levels in SW1353 cells. Cultures were exposed to increasing concentrations of recombinant human purified DKK1 protein (R&D Systems) for 2 hr, followed by SDS-PAGE and immunoblot analysis for total β-catenin (BD Transduction Laboratories), active β-catenin (anti-ABC, clone 8E7) dephosphorylated on Ser37 or Thr41 (Millipore) and β-actin (Sigma). B. SW1353 chondrosarcoma cells were transfected with either empty vector or DKK1-HA. Expression of tagged DKK1 was assessed by immunoblot analysis of lysates with an anti-HA antibody. Total β-catenin, active β-catenin, and β-actin were detected by Western blot as described above. C. DKK1 suppressed the TCF-reporter transcriptional activity in SW1353 cells. Cells were cotransfected with either TOP-FLASH or Fop-FLASH plasmid (Upstate Biotechnology), and the pRL-CMV plasmid (Upstate Biotechnology) encoding Renilla luciferase as an internal control for transfection efficiency. Luciferase activity was measured 48 h after transfection with the Dual-luciferase reporter assay system (Promega). The values represent the mean (±SD) of three independent experiments, and the ratio of the activity obtained with the wild-type TOP-FLASH plasmid to the activity observed with the mutant FOP-FLASH plasmid was shown.
(TIF)
