# Supplementary figures and images for: Elevated Levels of Dickkopf-1 Are Associated with β-Catenin Accumulation and Poor Prognosis in Patients with Chondrosarcoma
Source: PLoS One. 2014 Aug 21;9(8):e105414. doi: 10.1371/journal.pone.0105414 (PMC4140757; doi:10.1371/journal.pone.0105414)

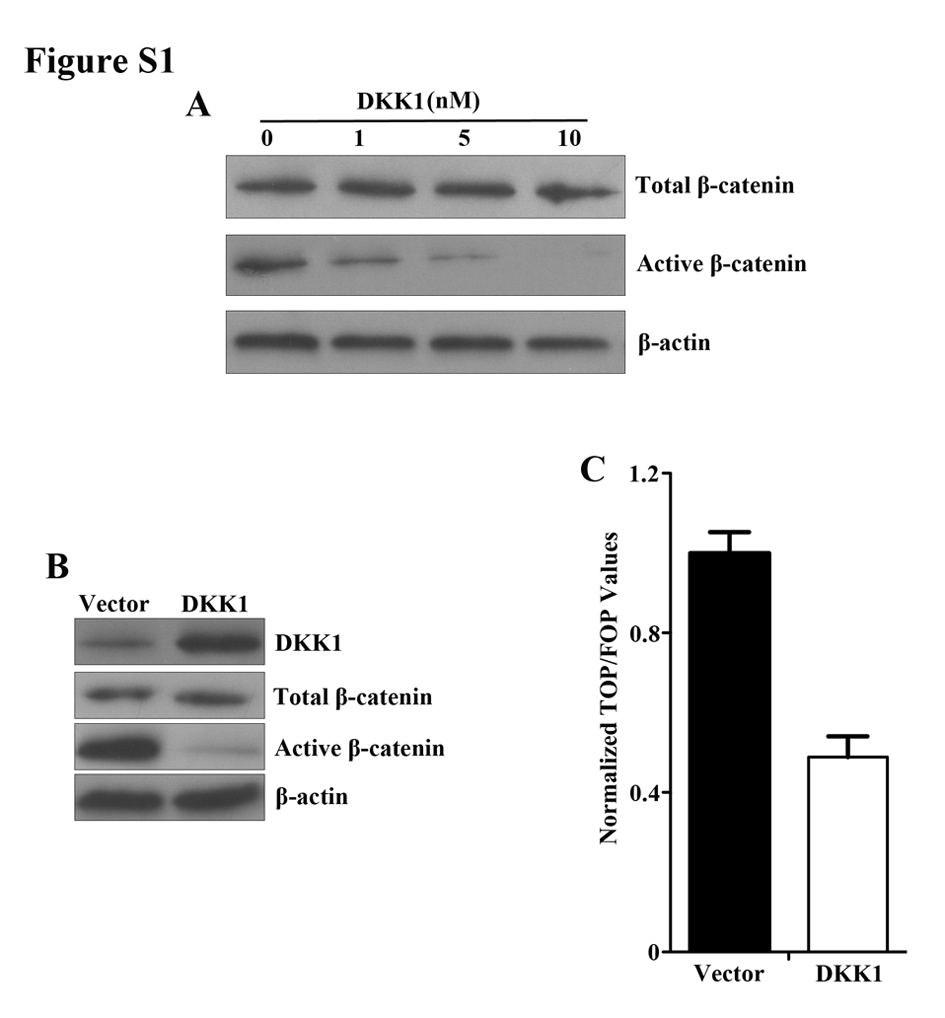

Supplement: Figure S1 — DKK1 inhibition of canonical Wnt/β-catenin signaling in human chondrosarcoma SW1353 cells. A. Soluble DKK1 inhibited active β-catenin levels in SW1353 cells. Cultures were exposed to increasing concentrations of recombinant human purified DKK1 protein (R&D Systems) for 2 hr, followed by SDS-PAGE and immunoblot analysis for total β-catenin (BD Transduction Laboratories), active β-catenin (anti-ABC, clone 8E7) dephosphorylated on Ser37 or Thr41 (Millipore) and β-actin (Sigma). B. SW1353 chondrosarcoma cells were transfected with either empty vector or DKK1-HA. Expression of tagged DKK1 was assessed by immunoblot analysis of lysates with an anti-HA antibody. Total β-catenin, active β-catenin, and β-actin were detected by Western blot as described above. C. DKK1 suppressed the TCF-reporter transcriptional activity in SW1353 cells. Cells were cotransfected with either TOP-FLASH or Fop-FLASH plasmid (Upstate Biotechnology), and the pRL-CMV plasmid (Upstate Biotechnology) encoding Renilla luciferase as an internal control for transfection efficiency. Luciferase activity was measured 48 h after transfection with the Dual-luciferase reporter assay system (Promega). The values represent the mean (±SD) of three independent experiments, and the ratio of the activity obtained with the wild-type TOP-FLASH plasmid to the activity observed with the mutant FOP-FLASH plasmid was shown. (TIF) [file pone.0105414.s001.tif]
